# The relationship between survival and socio-economic status for head and neck cancer in Canada

**DOI:** 10.1186/1916-0216-43-2

**Published:** 2014-01-14

**Authors:** James Ted McDonald, Stephanie Johnson-Obaseki, Euna Hwang, Chris Connell, Martin Corsten

**Affiliations:** 1 Department of Economics, University of New Brunswick, Fredericton, Canada; 2 Department of Otolaryngology-Head and Neck Surgery, University of Ottawa/The Ottawa Hospital, Suite S-3, 501 Smyth Road, Ottawa, ON K1H 8L6, Canada

**Keywords:** Head and neck cancer, Survival, Socioeconomic status, Income, Age at diagnosis, Human papillomavirus

## Abstract

**Background:**

Human papilloma virus (HPV) is emerging as the primary cause for some head and neck cancers. The objective of this study was to investigate the association between head and neck cancer (HNC) survival and socioeconomic status (SES) in Canada, and to investigate changes in the relationship between HNC survival and SES from 1992 to 2005.

**Methods:**

Cases were drawn from the Canadian Cancer Registry (1992–2005), and were categorized into three subsites: oropharynx, oral cavity, and “other” (hypopharynx, larynx, and nasopharynx). Demographic and socioeconomic information were extracted from the Canadian Census of Population data for the study period, which included three census years: 1991, 1996 and 2001. We linked cases to income quintiles (InQs) according to patients’ postal codes.

**Results:**

Overall survival, without controlling for smoking, for oropharyngeal cancer increased dramatically from 1992–2005 in Canada. This increase in survival for oropharynx cancer was eliminated by the introduction of controls for smoking. Survival for all head and neck cancer subsites was strongly correlated with SES, as measured by income quintile, with lower InQ’s having lower survival than higher. Lastly, the magnitude of the difference in survival between the highest and lowest income quintiles increased significantly over the time period studied for oropharynx cancer, but did not statistically significantly change for oral cavity cancer or other head and neck cancers.

**Conclusions:**

These data confirm a significant impact of socioeconomic deprivation on overall survival for head and neck cancers in Canada, and may provide indirect evidence that HPV-positive head and neck cancers are more common in higher socioeconomic groups.

## Background

Head and neck cancer (HNC) accounts for approximately 3% of all new malignancies in the United States, and is the fifth most common human cancer worldwide. In 2012 there were approximately 52,610 new cases in the United States, and 11,500 deaths from HNC [1]. Many cancers have been demonstrated to have worse survival in patients with lower socio-economic status [2]. The reasons for this relationship are multifactorial, and may include stage at presentation, education [3], access to health care services, diet, environmental exposures, and differential levels of tobacco and alcohol consumption. Booth et al examined the effects of median household income on survival for a number of cancers in Ontario Canada, including head and neck, and found a substantial gradient in survival between the highest and lowest income quintiles for all cancers studied [4].

A number of studies have examined the relationship between survival and socioeconomic status for HNCs in general and/or specific head and neck subsites. In 1999, Rachet et al. looked at survival for laryngeal cancer in England and Wales, and found that five-year survival was 17% lower (95% CI 12-22%) among men diagnosed in the most deprived group than those in the most affluent group [5]. This was the steepest socioeconomic gradient they found in all of the 20 common cancers they examined in this fashion. Chu et al examined Asian and Pacific Islanders in the United States using California Cancer Registry data from 1998–2007, and found that lower neighborhood SES was significantly associated with poorer disease specific and overall survival after adjusting for patient and tumor characteristics across all head and neck subsites [6]. Jovanovic-Andersen et al. also found lower survival with lower socioeconomic position as measured by a number of different parameters across head and neck subsites in Denmark [7]. For oropharyngeal cancer specifically, Reitzel et al. identified poor overall survival among patients from highly deprived neighborhoods relative to less deprived neighborhoods in the United States [8].

There has been some literature examining the survival – SES relationship for cancer in Canada as well. Boyd et al. examined all incident cases of cancer in Ontario using the Ontario Cancer Registry from 1987–1992 and compared this with the Surveillance, Epidemiology, and End Result (SEER) Database in the United States [9]. For HNC, they found a 7.4% difference (CI 3.1%-11.7%) between the highest income quintile and the lowest in Ontario, and a 13.7% difference (CI 11.1%-16.3%) in the United States. The same group published on SES effects in larynx cancer [10]. They again identified patients using the Ontario Cancer Registry, and used income quintiles based on median household income. They found no relationship between SES and outcome for supraglottic cancer, but there was an association identified for glottic cancer: the relative risk for patients in the lowest income quintile was 2.75 (CI 1.48-5.12) for cause-specific death and 1.90 (CI 1.24-2.93) for loco-regional failure.

Dramatic changes have been seen in the epidemiology of HNC, particularly oropharynx cancer, in the past two decades. These changes are related to the increase in the proportion of HNCs caused by human papilloma virus (HPV) [11]. HPV-related cancers are known to have increased survival compared to non-HPV-related cancers in the head and neck [12]. The survival for non-HPV-related cancers has been relatively stable. What is not known is whether the relationship between survival and SES is different for HPV-related cancers as compared to non-HPV-related cancers. Indirect evidence that the effect of SES on survival is different based on HPV status may come from identifying a difference in the SES-survival effect over time and/or identifying a different trend in oropharyngeal cancers, as opposed to other head and neck subsites.

Our group sought to investigate the SES/HNC survival relationship over the time period 1992–2005. To our knowledge, there has not been a Canadian study using nationwide Cancer Registry data to examine the relationship between HNC survival and SES. The goals of this study were two-fold: 1) to confirm the relationship between SES and HNC survival using Canadian Cancer Registry data; 2) to investigate the changes in the relationship between SES and HNC survival over time.

## Methods

Our data were drawn from three sources: the Canadian Cancer Registry (CCR) data file, the Vital Statistics (VS) data file, and the Canadian Census of Population. The CCR and VS are already linked at the individual level in the file provided by Statistics Canada to researchers through the Canadian Research Data Centre Network. Data were accessed in the New Brunswick Research Data Centre (NB-RDC) located at the University of New Brunswick in Fredericton, Canada. Our study complied with the University of New Brunswick Institutional Review Board ethics requirements, which do not necessitate an additional review for research projects using Statistics Canada data stored in the NB-RDC. The CCR data file contains patient demographic and tumor-specific data on each tumor recorded in provincial and territorial cancer registries from 1992 to 2007, inclusive. In order to be able to observe survival over a two-year period after diagnosis, we restrict attention to cancer cases diagnosed in 2005 or earlier. From the CCR data we obtained the sex of the individual, the age at which the tumor was diagnosed, and the individual’s six-digit postal code of residence. Data on demographic and socioeconomic information at the local area level were drawn from the Canadian Census of Population for the census years (CY) 1991, 1996, and 2001. The finest level of disaggregation at which census information is released by Statistics Canada is the Dissemination Area (DA), a small, stable geographic unit composed of one or more adjacent blocks, with a population of 400 to 700 persons. DAs cover the whole territory of Canada. For each DA and for each of the four CYs, information was collected on age for men and women, average household income, and proportion of adult residents with at least a university degree. Information at the DA level was computed for each CY and assigned to each individual in the CCR data as follows: cases diagnosed in 1992–1995 were associated with socioeconomic characteristics according to the 1991 Census; cases diagnosed in 1996–2000 were associated with data from the 1996 Census; and cases diagnosed in 2001–2005 were associated with data from the 2001 Census. The statistical tool PCCF + was used to map the six digit postal code available in the CCR data for each case of HNC to the relevant DA in which that postal code area was located.

Our key measure of interest was average household income. In order to control for wage and price differences across different regions, the income quintile (InQ) for each DA was defined relative to other DAs in the associated Census Division (CD), which Statistics Canada defines as a group of neighboring municipalities joined together for the purposes of regional planning and managing common services. In addition, although person-level data on smoking behavior is not available in the CCR, provincial smoking rates by education level were obtained for each CY from various waves of the Canadian Community Health Survey and the National Population Health Survey. For each DA in our sample and for each CY, a weighted average DA-level smoking rate was constructed using provincial smoking rates by education level weighted by the share of the adult population in each DA with that level of education. This composite smoking rate was included as an additional control variable in the regressions. A more detailed discussion of both CCR and Census data files and their linkage is available elsewhere [13].

The dependent variable was a binary variable that took the value 1 if the individual was still alive two years after the date of diagnosis and 0 if the individual was listed in the VS data as having died during that two-year period. Since we are examining survival over a two year period by restricting attention to cases diagnosed in 2005 and earlier, censoring is not an issue. We focus on two-year survival rather than five-year survival in order to have as long a time period as possible over which to investigate changes in the relationship between survival and SES. Statistical analysis employed logistic estimation methods, with the dependent variable expressed as a function of personal characteristics (sex, age at diagnosis, province of residence, CY of diagnosis) and DA-level characteristics (income quintile measured as a set of binary variables and weighted smoking rates). To allow trends over time in two-year survival to vary by SES, additional interactions of the CY indicator variables and the InQ variables were also included.

The CCR file developed by Statistics Canada for the Canadian research data centers provides ICD10 codes that are consistently defined over the whole sample period from 1992–2007. Of note, base of tongue was included in the oropharynx group and not in the oral cavity group. Regressions were estimated separately for each of three distinct subgroups of HNC, stratified by likelihood of causation by HPV, as in a prior study from our group [13]: i) cancers of the oropharynx (ICD-O-2/3 codes C019, C024, C051, C052, C090-103, C108-109, C140, and C142); ii) cancers of the oral cavity (C003-4, C020-C023, C029-C031, C039-C041, C049-50, C059-C062, and C069); and iii) cancers of the hypopharynx, larynx, and nasopharynx (C110-C139, C300, and C310-C329).

## Results

### Overall two-year survival

When examined without smoking controls, the overall two-year survival for oropharyngeal cancer increased dramatically between the 1991 CY and 2001 CY (OR 1.46, p = 0.00, CI 1.28-1.66). A similar but smaller magnitude increase in survival was seen for oral cavity cancer between the 1991 and 2001 CYs (OR 1.15, p = 0.02, CI 1.02-1.30), and there was no significant increase in survival for other HNCs (OR 1.05, p = 0.29, CI 0.96-1.15) (Table 1). The probability of survival for all three types of cancer was significantly lower when the age at diagnosis was older, and was lower for men for oropharyngeal and oral cavity cancers (Table 1). Once smoking controls were introduced, there was no longer a significant increase in survival for any cancer subsite over the duration of the study period (Oropharynx: OR 1.05, p = 0.64, CI 0.87-1.26; Oral cavity: OR 0.92, p = 0.92, CI 0.80-1.06; Other: 0.92, p = 0.22, CI 0.80-1.05). Higher smoking rates were also associated with lower incidence of survival for all three types of cancer, with a ten percentage point increase in smoking rate reducing the odds of survival by 31% for oropharynx (OR 0.69, p = 0.00, CI 0.58-0.81; Oral cavity: OR 0.78, p = 0.00, CI 0.68-0.89; Other: 0.86, p = 0.01, CI 0.76-0.96) (Table 2).

**Table 1:** Overall 2-year survival for head and neck cancers

	**Oropharyngeal**			**Oral cavity**			**Naso/hypo/laryngeal**		
	**n = 7022**			**n = 8541**			**n = 14665**		
	**OR**	**p-value**	**95% CI**	**OR**	**p-value**	**95% CI**	**OR**	**p-value**	**95% CI**
Male	0.748	0.000	(0.673-0.831)	0.703	0.000	(0.627-0.788)	1.015	0.717	(0.937-1.100)
CY 1991	1.000			1.000			1.000		
CY 1996	1.212	0.003	(1.068-1.374)	1.167	0.023	(1.022-1.334)	0.996	0.917	(0.919-1.079)
CY 2001	1.457	0.000	(1.277-1.663)	1.151	0.022	(1.021-1.298)	1.051	0.286	(0.959-1.152)
Age at diagnosis	0.952	0.000	(0.947-0.956)	0.962	0.000	(0.958-0.966)	0.969	0.000	(0.966-0.971)

**Table 2:** Overall 2-year survival for head and neck cancers with smoking controls

	**Oropharyngeal**			**Oral cavity**			**Naso/hypo/laryngeal**		
	**n = 7022**			**n = 8541**			**n = 14665**		
	**OR**	**p-value**	**95% CI**	**OR**	**p-value**	**95% CI**	**OR**	**p-value**	**95% CI**
Male	0.751	0.000	(0.676-0.833)	0.706	0.000	(0.629-0.791)	1.018	0.666	(0.939-1.103)
CY 1991	1.000			1.000				1.000	
CY 1996	0.968	0.691	(0.826-1.135)	1.002	0.975	(0.882-1.138)	0.907	0.073	(0.815-1.009)
CY 2001	1.045	0.643	(0.868-1.258)	0.922	0.254	(0.803-1.060)	0.917	0.216	(0.800-1.052)
Age at diagnosis	0.952	0.000	(0.947-0.956)	0.962	0.000	(0.959-0.966)	0.969	0.000	(0.966-0.972)
Smoking Rate	0.687	0.000	(0.582-0.810)	0.779	0.000	(0.684-0.887)	0.857	0.009	(0.764-0.960)

### SES status and survival

SES, as measured by income quintile, was strongly and significantly correlated with survival for all head and neck subsites. For oropharyngeal cancers, the odds ratio for survival for the lowest income quintile was 0.62 (p = 0.00, CI 0.52-0.74) compared to the highest income quintile (Table 3). There was a smooth statistically significant increase in survival across all five income quintiles. For oral cavity cancers, the odds ratio for survival for the lowest income quintile was 0.68 (p = 0.00, CI 0.56-0.82), whereas for all other head and neck subsites the odds ratio was 0.63 (p = 0.00, CI 0.56-0.70), with the same statistically significant pattern of increase with increasing income quintile (Table 3).

**Table 3:** Socio-economic status and 2-year survival for head and neck cancers

	**Oropharyngeal**			**Oral cavity**			**Naso/hypo/laryngeal**		
	**n = 7022**			**n = 8541**			**n = 14665**		
	**OR**	**p-value**	**95% CI**	**OR**	**p-value**	**95% CI**	**OR**	**p-value**	**95% CI**
Male	0.743	0.000	(0.669-0.826)	0.707	0.000	(0.631-0.792)	1.007	0.860	(0.928-1.094)
CY 1991	1.000			1.000			1.000		
CY 1996	1.025	0.750	(0.879-1.197)	1.054	0.443	(0.922-1.205)	0.958	0.439	(0.860-1.068)
CY 2001	1.147	0.133	(0.959-1.372)	1.002	0.981	(0.856-1.172)	1.009	0.902	(0.876-1.161)
Age at diagnosis	0.952	0.000	(0.948-0.957)	0.963	0.000	(0.959-0.966)	0.969	0.000	(0.966-0.972)
Smoking Rate	0.759	0.001	(0.645-0.892)	0.851	0.015	(0.747-0.970)	0.941	0.326	(0.833-1.063)
Highest InQ	1.000			1.000			1.000		
2nd InQ	0.826	0.087	(0.664-1.028)	0.892	0.271	(0.728-1.093)	0.863	0.008	(0.774-0.963)
Middle InQ	0.785	0.018	(0.642-0.960)	0.885	0.137	(0.753-1.040)	0.806	0.001	(0.706-0.920)
4th InQ	0.691	0.000	(0.578-0.826)	0.793	0.007	(0.671-0.937)	0.755	0.000	(0.671-0.849)
Lowest InQ	0.620	0.000	(0.520-0.739)	0.675	0.000	(0.558-0.818)	0.626	0.000	(0.558-0.701)

### Time trend for SES/survival relationship

In our final specification, we allowed changes in survival over time to vary by SES. In this specification the SES indicator variables correspond to the base census year 1991, while interactions of these SES indicator variables with CY indicator variables indicate whether the gap in survival incidence between higher and lower SES widens or narrows over time. For oropharynx cancers the odds ratio for survival of the lowest income quintile compared to the highest income quintile was significantly lower in 2001 than in 1991, as indicated by the interaction term for the lowest income quintile in 2001(OR 0.72, p = 0.05, CI 0.52-1.00) (Table 4). That is, the survival advantage for individuals in the highest InQ increased over the study period compared to individuals in the lowest InQ. Interaction terms for other quintiles in 2001 were also less than 1 but were not significant. For oral cavity cancers, interactions of income quintiles with 1996 and 2001 CYs were also less than 1 but were not significantly different from the difference in income quintiles at baseline 1991 CY. For oral cavity cancers, the odds ratio for the lowest income quintile in 2001 was 72% of that at baseline but was not significant (OR 0.72, p = 0.14, CI 0.46-1.12). For other HNC, the odds ratio for survival in 2001 for the lowest income quintile compared to the highest was also not significantly different from the pattern at baseline (OR 0.90, p = 0.56, CI 0.63-1.29) (Table 4).

**Table 4:** Changes in 2-year survival and socio-economic status for head and neck cancers

	**Oropharyngeal**			**Oral cavity**			**Naso/hypo/laryngeal**		
	**n = 7022**			**n = 8541**			**n = 14665**		
	**OR**	**p-value**	**95% CI**	**OR**	**p-value**	**95% CI**	**OR**	**p-value**	**95% CI**
CY 1991	1.000			1.000			1.000		
CY 1996	1.142	0.402	(0.837-1.558)	1.208	0.193	(0.909-1.607)	1.043	0.681	(0.852-1.277)
CY 2001	1.458	0.012	(1.087-1.954)	1.240	0.206	(0.889-1.731)	1.028	0.864	(0.753-1.403)
Age at	0.952	0.000	(0.948-0.957)	0.963	0.000	(0.959-0.966)	0.969	0.000	(0.966-0.971)
Smoking	0.763	0.001	(0.651-0.895)	0.858	0.020	(0.753-0.976)	0.945	0.374	(0.835-1.070)
Highest InQ	1.000			1.000					
2nd InQ	1.012	0.952	(0.697-1.468)	0.944	0.779	(0.631-1.411)	0.859	0.112	(0.712-1.036)
Middle InQ	0.850	0.351	(0.605-1.195)	1.024	0.872	(0.765-1.370)	0.793	0.036	(0.639-0.985)
4th InQ	0.754	0.037	(0.578-0.983)	0.906	0.592	(0.633-1.298)	0.825	0.071	(0.669-1.017)
Lowest InQ	0.767	0.063	(0.580-1.014)	0.830	0.346	(0.563-1.223)	0.688	0.002	(0.544-0.869)
2nd InQx96	0.725	0.128	(0.479-1.097)	0.959	0.839	(0.639-1.440)	0.962	0.811	(0.697-1.327)
2nd InQx01	0.795	0.267	(0.531-1.191)	0.879	0.595	(0.548-1.412)	1.070	0.625	(0.815-1.405)
Middle InQx96	1.060	0.797	(0.680-1.654)	0.856	0.408	(0.592-1.238)	0.960	0.772	(0.731-1.262)
Middle InQx01	0.757	0.118	(0.534-1.073)	0.765	0.152	(0.530-1.104)	1.113	0.549	(0.784-1.580)
4th InQx96	1.022	0.901	(0.727-1.437)	0.854	0.410	(0.588-1.242)	0.865	0.292	(0.659-1.133)
4th InQx01	0.764	0.156	(0.528-1.108)	0.793	0.342	(0.492-1.279)	0.904	0.549	(0.649-1.259)
Lowest InQx96	0.768	0.143	(0.540-1.093)	0.774	0.236	(0.507-1.182)	0.859	0.269	(0.655-1.125)
Lowest InQx01	0.722	0.050	(0.521-1.000)	0.717	0.139	(0.461-1.115)	0.899	0.559	(0.628-1.285)

## Discussion

Our study showed that survival for oropharynx cancer increased dramatically in Canada from the 1991 to 2001 census years, reflecting cancers diagnosed between 1992 and 2005 (OR 1.46). Survival for oral cavity cancers also increased, but to a lesser extent (OR 1.15). Survival did not significantly change for cancers in other head and neck subsites (OR 1.05). After controlling for smoking rates, this increase in survival for oropharyngeal and oral cavity cancers over the time period was no longer present. We feel there are two likely explanations for this phenomenon. First, this data was measuring all cause survival rather than disease specific survival. It seems likely that nonsmokers would have improved all cause survival compared with smokers, independent of their diagnosis of cancer. Second, there is a strong correlation between improved survival from oropharyngeal cancer and smoking status; that is, it has been demonstrated that the improved survival in HPV-positive oropharynx cancers is principally seen in nonsmokers [12,14]. Thus, the evidence for a survival benefit secondary to HPV-positivity might be eliminated once smoking controls are added.

Our second finding was that socioeconomic status, as measured by income quintile, was significantly correlated with survival. Individuals in the lowest income quintile had an odds ratio for overall survival of 0.62 compared to the highest income quintile for oropharyngeal cancer; the odds ratio was 0.68 for oral cavity cancer, and 0.63 for other HNCs. These data indicate that even in a country with universal healthcare, socioeconomic disparities have a substantial impact on survival. This confirms similar findings in other studies of the Canadian population [4,15].

The third and perhaps most interesting finding from these data relates to the time trend in the impact of socioeconomic status on survival in these cancers. The gap in survival between the highest and lowest income quintiles increased significantly over the time period studied for oropharyngeal cancer (the odds ratio for the lowest income quintile compared to the highest in the 2001 CY was only 72% of the equivalent odds ratio in 1991. (p = 0.05). There was no statistically significant difference in the survival gap for oral cavity cancers.

One possible explanation for this difference in time trend relates to the relationship between HPV-positive tumors and SES. To date, there has been no direct evidence to suggest whether HPV-positive HNCs are more common in high or low SES individuals. The widening gap in survival between the highest and lowest income quintile for oropharyngeal cancer in our study over time may serve as indirect evidence that HPV-positive HNCs may be more common in higher SES groups. This is potentially confirmed by the absence of such a time trend in cancers that are not as associated with HPV as oropharynx cancer related to HPV (oral cavity, nasopharynx, hypopharynx, and larynx). This potential association between higher socioeconomic status and HPV-positive HNCs should be investigated in future studies.

Our study had several limitations. First, no direct information about HPV-positivity in tumors was available, making any inference about the influence of HPV on the SES-survival relationship an indirect one. Second, we did not possess individual-level income data, and thus used DA-level information on household income as a substitute, which is less precise than individual-level information. Furthermore, while household income reflects income from all sources, including pensions and investment income, SES could be measured more accurately with individual-level information on other determinants of SES such as education level and occupation. Income alone may be biased in favor of current SES rather than long-run SES. Finally, there was not sufficient staging information available in the CCR dataset to allow for us to control for stage at presentation by sub-site. As a result, inferences on differential survival by HNC sub-site assume that stage at presentation is not different for the different sub-sites.

## Conclusions

Overall survival, without controlling for smoking, for oropharyngeal cancer increased dramatically from 1992–2005 in Canada. A smaller magnitude increase in survival was seen for oral cavity cancer, and no change was seen for cancer in other head and neck subsites. When we controlled for smoking, the increase in survival for oropharynx and oral cavity cancer was eliminated. Survival for all HNC subsites was strongly correlated with SES, as measured by income quintile, with lower InQ’s having lower survival than higher. Lastly, after controlling for smoking, the magnitude of the difference in survival between the highest and lowest income quintiles increased significantly over the time period studied for oropharynx cancer, but did not statistically significantly change for oral cavity cancer or other HNCs. These data confirm a significant impact of socioeconomic deprivation on overall survival for HNCs in Canada, and may provide indirect evidence that HPV-positive HNCs are more common in higher socioeconomic groups.

## Competing interests

The authors declare that they have no competing interests.

## Authors’ contributions

SJ-O - paper idea, and write up. JTM and CC - data collection and analysis. EH - write up. MC - ideas and write up. All authors read and approved the final manuscript.
